# Driving the future of value-based healthcare in the Gulf Cooperation Council: a roadmap for achieving sustainable access to specialty pharmaceuticals

**DOI:** 10.3389/fpubh.2025.1667846

**Published:** 2025-11-05

**Authors:** Anas Hamad, Ahmed Al-Jedai, Rita Ojeil, Abdulrazaq Sheikh Al-Jazairi, Adel AlAssy, Yazed S. AlRuthia, Waiel Al Naeem, Hajer Almudaiheem, Mouza Alsaadi, Nada Alagil, Lina Wahba, Abdulmohsin Marghalani, Amna Al Hashar, Abdullah O. AlShehry, Sana Alblooshi, Ibtisam Alharbi, Marleine Bejjani Moukarzel, Sara Albalushi, Ahmed M. El-Sheashaey, Mohammed A. Aseeri, Farid Alenezy, Fathea Adheir, Abdulrahman Aloumi, Rehab Alnoaimi, Khalid A. Alnaqbi

**Affiliations:** ^1^Pharmacy Department, National Center for Cancer Care and Research, Hamad Medical Corporation, Doha, Qatar; ^2^Colleges of Medicine and Pharmacy, Alfaisal University, Riyadh, Saudi Arabia; ^3^Life Sciences Solution, PDC-CRO, Dubai, United Arab Emirates; ^4^Clinical Trials Transformation Initiative Division, King Faisal Specialist Hospital and Research Centre, Riyadh, Saudi Arabia; ^5^Pharmaceuticals Supply Chain Management, M42 Health, Abu Dhabi, United Arab Emirates; ^6^Department of Clinical Pharmacy, College of Pharmacy, Riyadh, Saudi Arabia; ^7^Department of Pharmacy, Sheikh Khalifa Medical City, Abu Dhabi, United Arab Emirates; ^8^Clinical Pharmacy, The Saudi Society of Clinical Pharmacy, Riyadh, Saudi Arabia; ^9^Department of Health Economics and Insurance Policies, Dubai Health Authority, Dubai, United Arab Emirates; ^10^Clinical Pharmacy, Council of Health Insurance, Riyadh, Saudi Arabia; ^11^Adult Oncology Unit and Pharmacy Department, Tawam Hospital, Abu Dhabi, United Arab Emirates; ^12^Pharmaceutical Care Division, King Faisal Specialist Hospital and Research Centre, Jeddah, Saudi Arabia; ^13^Sultan Qaboos Comprehensive Cancer Center, University Medical City, Seeb, Oman; ^14^Pharmacy Services Administration, King Fahad Medical City, Riyadh, Saudi Arabia; ^15^Department of Pharmacy, Tawam Hospital, SEHA-Abu Dhabi Health Services, Al Ain, United Arab Emirates; ^16^Pharmacoeconomic Centre, King Fahd Ahmed Forces Hospital, Jeddah, Saudi Arabia; ^17^Department of Pharmacy, Sheikh Shakhbout Medical City, Abu Dhabi, United Arab Emirates; ^18^Pharmaceutical Care Department, Directorate General of Medical Supplies, Ministry of Health, Muscat, Oman; ^19^Pharmacy Department, Kuwait Cancer Control Centre, Al Sabah Specialized Medical District, Ministry of Health, Shuwaikh, Kuwait; ^20^Clinical Pharmacy, King Saud bin Abdulaziz University for Health Sciences, Jeddah, Saudi Arabia; ^21^Pharmacy Department, Sidra Medicine, Ar-Rayyan, Qatar; ^22^Pricing Department, Pharmaceutical and Herbal Medicine Registration and Control Administration, Ministry of Health, Kuwait City, Kuwait; ^23^Procurement, SHIFA National Medical Stores, Manama, Bahrain; ^24^Rheumatology Division, Sheikh Tahnoon Medical City, SEHA/Pure Health, Al Ain, United Arab Emirates

**Keywords:** Gulf Cooperation Council, value-based healthcare, specialty drugs, consensus development, managed entry agreement

## Abstract

Gulf Cooperation Council (GCC) countries are undergoing a critical transformation in their healthcare systems. This empowers them to address the rising burden of complex diseases, including rare diseases, cancer, neurological disorders, and immunological illnesses, which involve a high cost of therapy. A strategic shift from volume- to value-based healthcare (VBH) emphasizes sustainability, enhanced accessibility, and improved health outcomes through innovation. GCC’s healthcare is marked by universal coverage and a shifting landscape of public-private partnerships. Rising pharmaceutical costs, especially for specialty drugs, continue to challenge budget sustainability. VBH offers a strategy to align healthcare expenditure with patient outcomes. This framework is supported by global and regional models such as managed entry agreements (MEAs), multi-criteria decision analysis, and real-world evidence (RWE). These models provide guidance for reimbursement strategies and support decision-making regarding high-value treatments. The GCC nations are also progressing towards policy discussion, but face challenges related to infrastructure, regulation, and workforce capacity. The Department of Health (DOH) in Abu Dhabi, which is a governmental health authority in the United Arab Emirates, has officially established a dedicated HTA unit to evaluate and assess new health technologies for evidence-informed decision making. This review highlights specialty care priorities and proposes target strategies such as expanding genetic databases, implementing screening programs, and establishing risk-sharing agreements to improve affordability, particularly for rare diseases. A consensus-driven phased roadmap for GCC-wide VBH adoption is recommended. This includes a focus on MEAs and patient-reported outcome measures, mid-term harmonization of health technology assessments (HTA) and RWE development as well as long-term establishment of digital ecosystems and value-based pricing platforms. Equitable and collaborative policies will be essential to achieving sustainable and inclusive healthcare systems across the GCC.

## 1 Introduction

Healthcare expenditure across the Gulf Cooperation Council (GCC) countries ranges from 2.6 to 6% of the gross domestic product (GDP); however, these figures may underestimate the actual expenditure amid rapid health system reforms aimed at improving access, efficiency, and quality (1, 2). Despite universal healthcare coverage for citizens and widespread private insurance for expatriates, considerable variations exist in spending patterns, regulatory structures, and pharmaceutical procurement mechanisms across the region (2).

The GCC countries are grappling with a high burden of non-communicable diseases (NCDs), including diabetes, cardiovascular diseases, obesity, and cancer, with prevalence rates significantly exceeding global averages. In parallel, high rates of consanguineous marriages contribute to the prevalence of genetic disorders such as thalassemia and sickle cell disease (SCD), with Bahrain showing an exceptionally high SCD prevalence at 12%. While countries like the United Arab Emirates (UAE) have reported relative success in reducing the incidence of β-thalassemia and SCD through preventive programs, cases of other severe genetic disorders such as Duchenne muscular dystrophy and spinal muscular atrophy are also on the rise in the Gulf (2–4).

Despite improvements in overall health indicators, life expectancy in most GCC countries remains slightly below that of some Organization for Economic Co-operation and Development (OECD) nations. Additionally, while infant and maternal mortality rates are better than global averages, they still lag behind those observed in countries like France and the United Kingdom. This highlights the urgent need for more robust, equitable, and sustainable healthcare interventions across the region (2, 5).

One of the major limitations for effective management of these conditions is the limited accessibility and availability of specialty or orphan drugs. These high-cost medications are specifically designed to treat rare, chronic, or complicated health conditions and often require special handling, administration, and monitoring (6, 7). This creates significant barriers to timely and equitable patient care requiring specialty drugs like gene therapies, biologics, and precision oncology drugs. These transformative therapies also present sustainability challenges due to their clinical complexity, pricing, and the need for specialized infrastructure (2, 8).

Faced with rapid advancements in treatments and rising healthcare costs, even the wealthiest nations struggle to ensure sustainable access to new medicines. Healthcare payers are often required to make early reimbursement decisions based on limited or uncertain evidence, while balancing equity across therapeutic areas (9).

Public healthcare systems in the GCC are under increasing pressure to expand access to innovative therapies while maintaining a balance between increasing access and fiscal responsibility, particularly in contexts where health technology assessment (HTA) capacity is limited and data systems are fragmented. For example, pharmaceutical expenditure as a share of total health spending ranges from 11% in Qatar to 21.7% in Oman (2).

HTA plays a significant role in supporting evidence-informed decisions on the adoption and reimbursement of health technologies. It systematically evaluates the clinical, economic, and social value of interventions. In parallel, VBH offers a broader strategic framework aimed at optimizing health outcomes relative to cost. It does so by aligning reimbursement mechanisms with real-world performance. Thereby, it incentivizes efficient resource use and supports the transition from a volume-based to outcome-driven care model (10).

While HTA and VBH are conceptually distinct, they are highly complementary. HTA informs what should be covered, while VBH informs how care should be delivered and rewarded (Table 1). Notably, recent publications from the region, highlight growing interest in formalizing HTA structures and exploring value-based purchasing models for high-cost therapies (1, 11–13). This review explores the readiness of GCC countries to adopt VBH principles, with a particular focus on high-specialty pharmaceuticals. We examine current policy landscapes, identify key enablers such as managed entry agreements (MEAs) and real world evidence (RWE). We also propose a tailored roadmap for the region’s healthcare context. The objective of the present paper is to evaluate the readiness, barriers, and implementation pathways for VBH in high-cost therapeutic areas in the GCC.

**Table 1:** Comparison between health technology assessment (HTA) and value-based healthcare (VBH) (130–132).

Aspect	Health technology assessment	Value-based healthcare
Scope	Evaluates technologies	Redesigns systems of care
Focus	Evidence-based approval and pricing	Outcomes-based care delivery
Main question	“Is this technology worth paying for?”	“Are we delivering the best outcomes for the cost?”
Users	Policymakers, payers, insurers	Health systems, providers, policymakers
Typical output	Reimbursement recommendation	New care models, key performance indicators, payment reforms

## 2 Methods

### 2.1 Study design

This manuscript employs a narrative review design to explore VBH implementation across GCC countries. It synthesizes insights from academic literature, policy frameworks, and global initiatives. The focus is on key VBH domains such as outcome-based reimbursement, digital health, access to high-cost therapies, specialty care models, and stakeholder engagement. Emerging priorities like rare diseases, cancer, immunology, and neurology were emphasized due to their growing regional impact. Additionally, this review was informed by insights from multidisciplinary key opinion leaders across the GCC. Their clinical, policy, and strategic expertise added depth to the analysis of implementation challenges and opportunities.

### 2.2 Literature search strategy

#### 2.2.1 Phase 1: scoping literature review

To collect relevant literature, structured searches were conducted across electronic databases, including PubMed, Scopus, Web of Science, and Google Scholar. Additional grey literature was sourced from official GCC government portals, MoH publications, national vision strategies (e.g., Saudi Vision 2030, UAE Health Sector Transformation Agenda). The reports from global health organizations such as the World Health Organization (WHO), OECD, and World Bank were also included.

#### 2.2.2 Phase 2: expert insights

**Figure 1:**
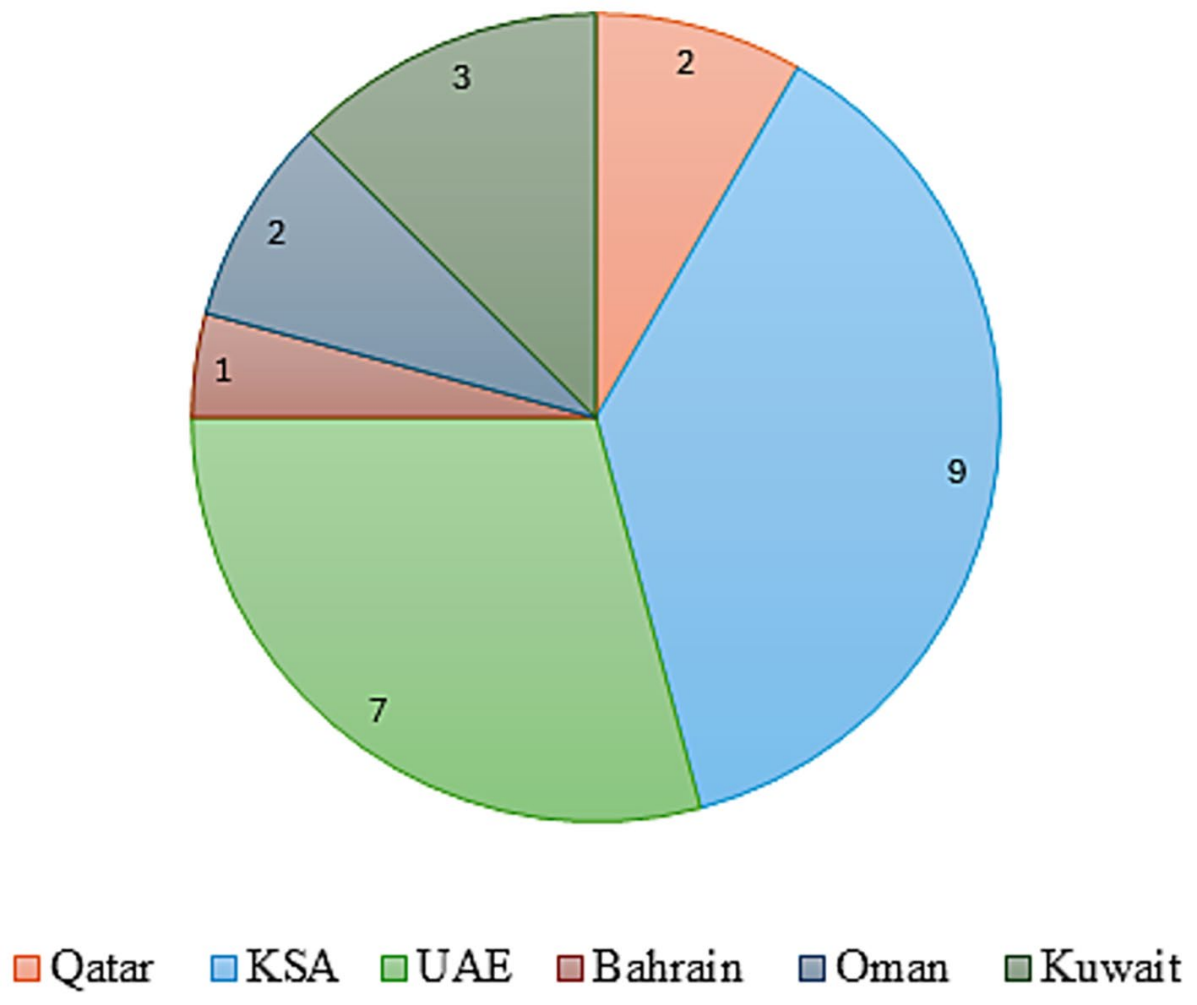
Countrywide distribution of consensus experts.

To assess the landscape and preparedness for implementing VBH across GCC countries, particularly in the context of specialty pharmaceuticals, a qualitative focus group discussion was convened. This session, held in the UAE on April 11–12, 2025, engaged 24 key opinion leaders from the GCC countries. Figure 1 gives a countrywide distribution of the experts. The group included representatives from the pharmaceutical and healthcare sectors, clinical practitioners, policy advisors, and officials from government health agencies. Experts from different GCC nations were engaged. Their identification and selection were aimed at ensuring broad regional and inter-disciplinary representation. The panel was designed to reflect balanced representation across geography (6 GCC countries), professional backgrounds (clinical, academic, regulatory, and policy), and institutional affiliations (government, hospital, and academic). This minimized the risk of dominance by any single discipline or country and strengthened the representativeness of the consensus process.

Experts were selected based on the following inclusion criteria:

1. They serve in an advisory capacity within public healthcare systems, focusing on specialty pharmaceuticals and VBH.
2. They hold senior roles in regulatory bodies such as the MoH or local health authorities and bring substantial field experience.
3. They are associated with leading academic institutions, possess a strong publication record in public health and VBH, and have provided consultative input to regulatory agencies in their respective countries.

To minimize potential bias and ensure methodological rigor, the experts also declared potential conflicts of interest prior to participation; no material conflicts were identified.

### 2.3 Search terms and inclusion criteria

The search strategy used combinations of the following terms: “Value-based healthcare,” “VBH,” “VBHC,” “health system reform,” “health technology assessment,” “health financing,” “bundled payments,” “outcome-based reimbursement,” “rare diseases,” “orphan drugs,” “immunology,” “neurology,” “oncology,” “cancer,” “implementation strategies,” “digital health,” “real-world evidence,” “real-world data,” “Gulf Cooperation Council,” and individual country names (“Saudi Arabia,” “Kingdom of Saudi Arabia,” “United Arab Emirates,” “UAE,” “Qatar,” “Oman,” “Kuwait,” and “Bahrain”). Boolean operators (AND/OR/NOT) were used to refine results, with filters applied to include publications from 2010 to 2025 and only English-language sources. Identified documents were reviewed and thematically analyzed to capture trends, opportunities, and systemic challenges relevant to the implementation of VBH within the GCC context.

### 2.4 Data screening and analysis

The identified documents were reviewed and analyzed to identify trends, opportunities, and systemic challenges relevant to VBH implementation within the GCC context. Expert insights provided valuable context and depth to the interpretation of the findings.

A structured meeting guide containing 17 core questions was developed following an in-depth review of existing literature. It addressed a broad range of themes, including clinical and economic hurdles in value-based evaluations for specialty drugs, and ethical and operational considerations in managing orphan and rare diseases. It also examined the adoption of novel treatments in fields including oncology, hematology, immunology, neurology, and rare diseases. The discussion framework also explored current insurance trends and reimbursement mechanisms aimed at improving the use and accessibility of high-cost therapies.

### 2.5 Consensus development process

On April 12, 2025, the summit adopted the RAND/UCLA appropriate methodology to build expert consensus on strategic priorities. The process unfolded in structured phases comprising presentation of evidence and regional context, deliberate discussions aligned with six strategic pillars, thematic prioritization, and plenary consensus recommendations. Plenary talks provided structured overviews of MEAs, outcomes-based agreements, reformation of the Pharmacy and Therapeutics Committee (P&T) process, and formulary frameworks across GCC and global settings. The six strategic pillars along which the discussions were focused comprised policy and regulatory infrastructure, health digitalization, patient outcome measurement, RWE generation, HTA or multi-criteria decision analysis (MCDA) utilization, and innovative contracting. A thematic content analysis was employed to analyze both literature and summit insights, categorizing responses into challenges, solutions, and collaboration and coordination among GCC countries and alignment with global practices.

**Figure 2:**
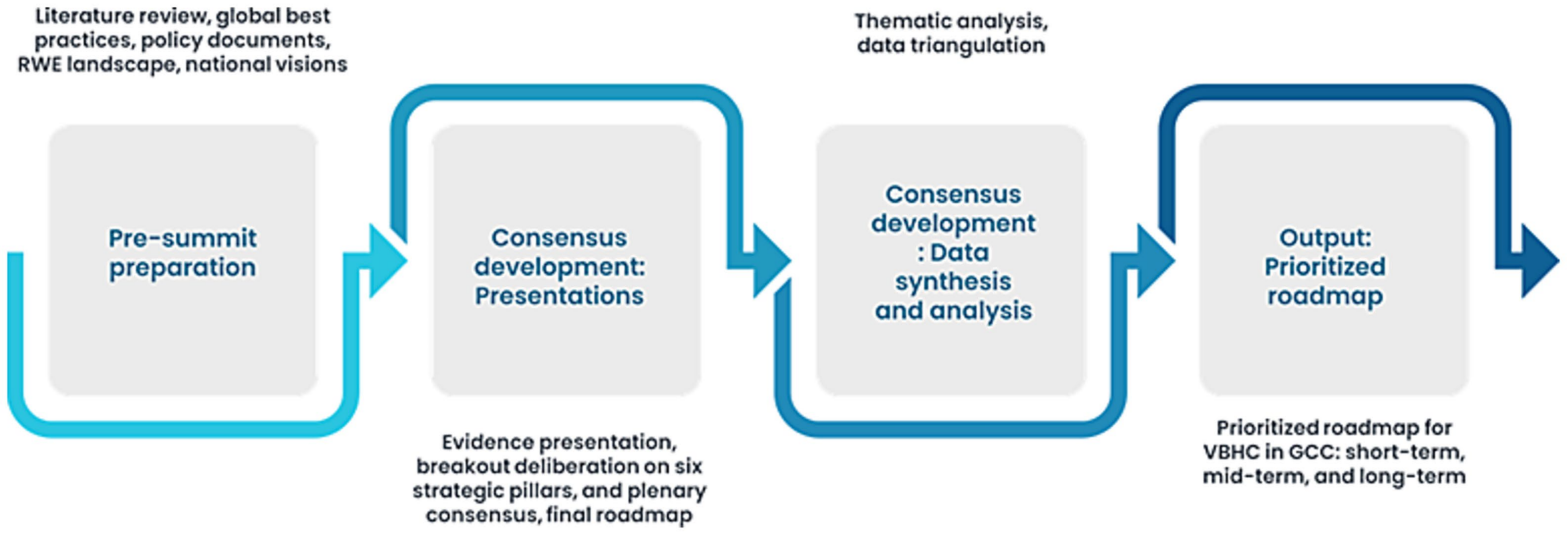
Framework to develop consensus.

The expert workshop data (notes, transcripts, and poll results) were categorized using the same thematic framework applied to the literature review. Responses were further delineated into challenges, solutions, and opportunities for collaboration. Themes were validated against existing policy frameworks and global literature to ensure robustness. Consensus was achieved when ≥80% of experts agreed with the identified themes, and structured voting rounds quantified levels of agreement. Insights were validated across the literature, policy documents, and expert recommendations. Figure 2 depicts the GCC VBH consensus framework.

Thematic coding of transcripts and notes was conducted independently by two researchers to ensure consistency. Codes were iteratively refined into categories and themes through consensus meetings. Validation was achieved by triangulating expert responses with literature findings and policy documents, while discrepancies were resolved through discussion.

## 3 Results

### 3.1 Regional overview

The healthcare landscape in the GCC countries is being reshaped by the rising burden of complex, high-cost medical conditions that demand specialized care and advanced therapeutic interventions. These conditions are emerging as critical cost drivers within national healthcare budgets (2).

The management of these conditions, including NCDs, genetic disorders, autoimmune diseases, and neurologic diseases, relies heavily on specialty pharmaceuticals such as biologics, gene therapies, and precision drugs. These therapies, while clinically transformative, are associated with substantial financial and operational demands due to their high cost, and complex administration requirements. They also require continuous outcome monitoring and specialized support services. The higher prevalence of genetic and rare diseases in the region further compounds this challenge (3, 14).

**Figure 3:**
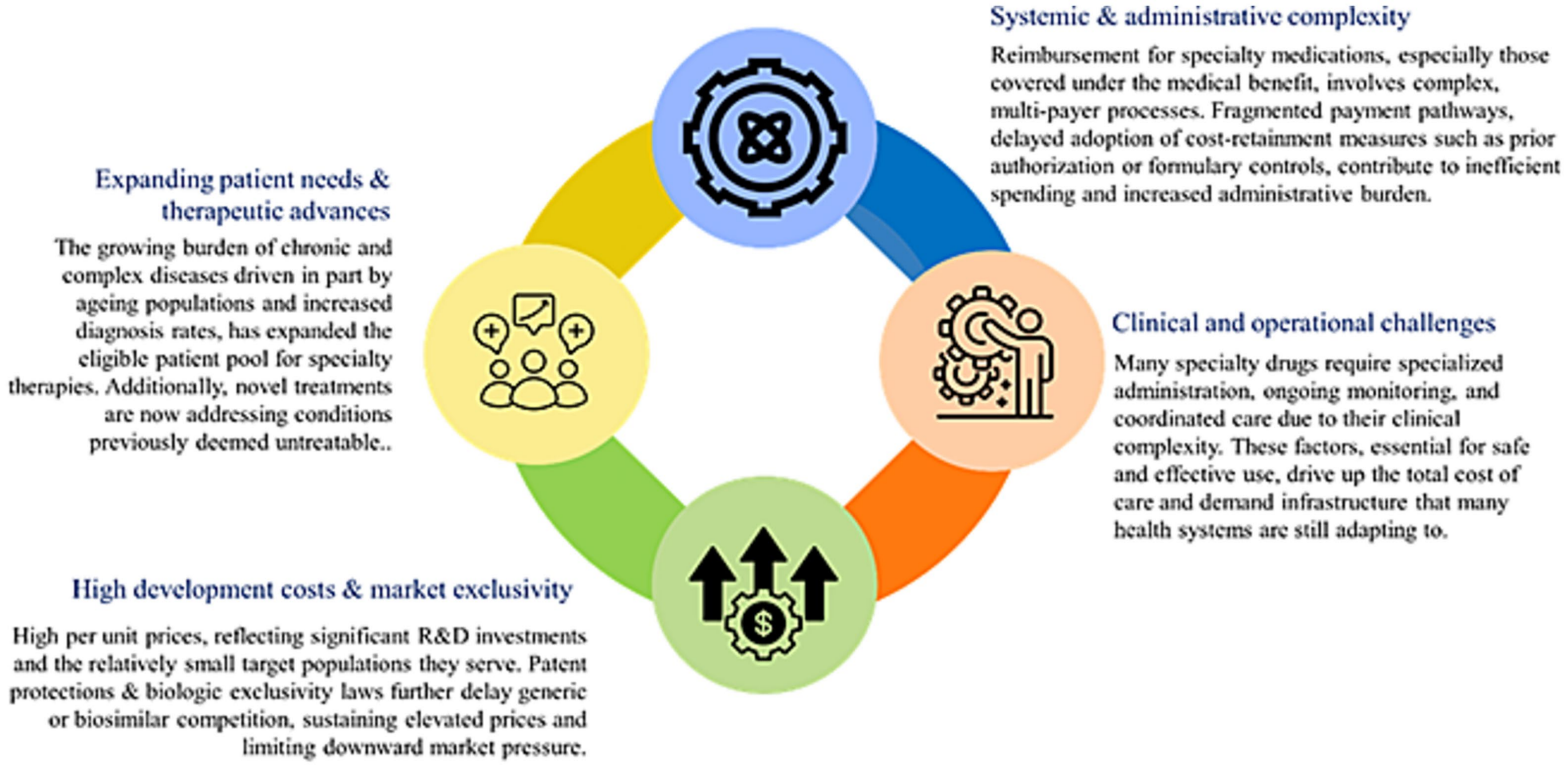
Key drivers of specialty drug expenditure.

In 2019, NCDs cost GCC countries an estimated US $50 billion, about 3.3% of regional GDP; of this, 60% (US $30 billion) is spent on direct treatment of diabetes, cancer, cardiovascular, and respiratory diseases (15). These costs are largely driven by hospital-based care and are expected to rise with aging populations. The additional indirect costs stem from lost productivity, reduced educational investment, and unpaid caregiving, placing further strain on health spending and pharmaceutical expenditures across the region (16). Although the GCC’s age-standardized cancer incidence is <100 per 100,000 which is about one-third of Western countries, the absolute number of cases is rising rapidly, with breast, colorectal, and thyroid cancers accounting for about 40% of new cases (17). This growing burden is projected to more than double by 2040, translating into escalating healthcare costs, workforce pressures, and productivity losses, thereby posing a significant economic challenge for GCC countries despite their currently lower incidence rates (17). Figure 3 shows the key drivers responsible for the escalating costs of specialty drugs.

### 3.2 Research and development

Current healthcare financing structures across the GCC countries are predominantly government-funded. Public healthcare systems cover the cost of pharmaceuticals and medical devices through the MoH and affiliated institutions such as military hospitals and medical cities, as well as local health authorities. However, procurement mechanisms differ by country. For example, in the KSA, public healthcare institutions are mandated to source medications and medical devices through a centralized tendering process via National Unified Procurement Company (NUPCO), whereas private providers have greater procurement flexibility, subject to Saudi Food and Drug Authority (SFDA) approval. The UAE E does not have a single procuring body for medicines and medical devices. Besides, the KSA and UAE take part in the Gulf Joint Procurement Program. This facilitates the collective procurement of medications, vaccines, and medical devices for select public health institutions (2).

### 3.3 Country snapshots

The GCC countries are progressively transitioning from volume-based to VBH models, driven by the increasing healthcare expenditures and a growing commitment to global quality healthcare standards. However, VBH implementation remains uneven, particularly in high-cost, high-burden therapeutic areas such as oncology and rare diseases, where precision and longitudinal outcome tracking are essential (2, 18).

#### 3.3.1 Kingdom of Saudi Arabia

The KSA has made notable progress under its Vision 2030 health transformation agenda, which includes key reforms such as digital health integration and a centralized procurement strategy (14, 18, 19). Oncology is a national priority under the cancer control strategy. However, policies for rare disease management remain underdeveloped and inconsistently applied (20, 21).

The vision outlines a comprehensive health reform agenda focused on preventive care, digital health, and emergency preparedness, public health campaigns promoting nutrition, physical activity, and smoking cessation, alongside school- and community-based programs. This aims to reduce the long-term burden of NCDs, offering a replicable model for other GCC countries (22, 23). The integration of digital tools such as electronic health records (EHRs), telemedicine, and artificial intelligence (AI) diagnostics has improved system efficiency and access to specialist care (24, 25). A review of Saudi MOH data showed rapid expansion of telemedicine during COVID-19, with 12 mobile apps (three launched during the pandemic) supporting millions of virtual consultations. “Sehhaty” was central, while “Mawid,” “Seha,” and the 937 hotline services saw dramatic increases in users and service volumes, reflecting a nationwide shift to digital healthcare (24).

Among GCC countries, KSA bore the largest share with 45% of NCDs related deaths, 49% of years of life lost, and 60% of economic losses (16). Therefore, KSA’s collaboration with the WHO to strengthen health emergency infrastructure provides a strategic framework for enhancing regional resilience (26).

#### 3.3.2 United Arab Emirates

The UAE’s approach to VBH emphasizes outcome-oriented and patient-centered care, underpinned by effective communication and technological integration. These reforms aim to improve clinical outcomes, enhance patient engagement, and reduce inefficiencies in healthcare delivery. While disparities in quality and care coordination persist, policymakers have acknowledged these challenges. They have demonstrated a strong commitment to embedding VBH principles into the national health strategy (19, 25).

The Department of Health has introduced an HTA roadmap to support universal health coverage. It focuses on making healthcare decisions more evidence-based and cost-effective by involving all stakeholders, including policymakers, providers, patients, and industry. The plan includes building local expertise, establishing governance, and promoting transparency through a phased five-year implementation. This initiative aims to create a more sustainable and inclusive healthcare system in the emirate (27).

Digital transformation is pivotal to advancing cancer care in the UAE, with e-health platforms, digital registries, and tele-oncology enabling earlier detection, better coordination, and more equitable access. Strengthening digital infrastructure and workforce training will be essential to deliver high-quality, affordable, and patient-centered cancer services. The UAE National Cancer Registry (UAE-NCR) was established to systematically collect, analyze, and report cancer incidence and mortality data across the country. The registry plays a crucial role in shaping national cancer control strategies, enabling early detection, screening programs, and resource allocation. It also highlights disparities in cancer incidence between UAE nationals and expatriates, and between genders (28).

#### 3.3.3 Qatar

Qatar demonstrates strong institutional readiness for VBH, owing to a highly centralized healthcare system and advanced digital infrastructure. The national cancer strategy supports robust outcome monitoring. However, care for rare diseases remains fragmented across the public and private sectors, limiting cohesive policy implementation (29). Qatar’s national health strategy focused on delivering person-centered care, improving healthcare outcomes, and ensuring the sustainability of the health system (22). The strategy promoted the integration of services, prioritization of prevention, and enhancement of care quality and efficiency, principles closely aligned with VBH. It also underscores the importance of data-driven decision-making and the use of health technology to improve patient outcomes and resource allocation. These initiatives reflect Qatar’s commitment to shifting from volume to value in healthcare delivery. Qatar’s national cancer framework builds on the earlier national cancer strategy to enhance cancer care by aligning services with patients’ needs. It outlines nine domains with defined activities and success metrics to guide implementation and evaluate outcomes (30).

#### 3.3.4 Kuwait, Oman, and Bahrain

These countries are in the early phases of VBH adoption, with policy discussions gaining momentum but limited operational structures currently in place. Their ongoing reforms primarily target improvements in quality of care and patient value, although standardized frameworks and outcome-based reimbursement models are still nascent (31).

Kuwait’s Vision 2035 “New Kuwait,” explicitly prioritizes improving healthcare quality, major hospital expansion, and digital transformation, giving VBH strong strategic alignment. The government has allocated a substantial healthcare budget and launched infrastructure expansion projects (new hospitals, partnerships with international centers) (32).

Oman Vision 2040 places health as a national priority, and the government is advancing initiatives to attract private investment and strengthen health sector capability helpful for mixed public/private VBH models (33).

Bahrain’s health sector is compact and well-regulated through the National Health Regulatory Authority (NHRA). It has piloted insurance reforms and has a mixed provider landscape, making it a strong testbed for value-based care. The National Health Plan and Economic Vision 2030 stress quality, sustainability, and data-driven outcomes, aligning with VBH principles. It has piloted insurance reforms and has a mixed provider landscape, making it a strong testbed for value-based care (34).

### 3.4 Specialty areas

Specialty care refers to advanced medical services provided by healthcare professionals with expertise in specific areas of medicine, such as cardiology, oncology, neurology, immunology, and rare diseases. These services go beyond general or primary care, focusing on the diagnosis, treatment, and management of complex or chronic conditions that require specialized knowledge, equipment, and facilities (35). The KSA is prioritizing specialty care in key areas, including oncology, neurology, immunology and rare diseases. The focus is on enhancing access to advanced treatments, expanding specialized facilities, and investing in workforce development to meet the growing demand for complex, high-quality care (8, 36). Gene therapy is a groundbreaking treatment for many rare diseases, targeting the root genetic defect. However, despite its potential, it remains one of the costliest medical interventions today (37, 38).

The KSA has also taken a significant step toward enhancing specialty care by implementing a specialty pharmacy model within a government-supported tertiary care hospital. The specialty pharmacy is designed to provide patient-centered services. This includes specialized counseling, streamlined dispensing, and improved medication access. The initiative led to a notable increase in patient and healthcare provider satisfaction, a 52% reduction in pharmacy waiting times. Further, a significant improvement in patient adherence to specialty medications (from 73.6 to 85.6%) is also observed (39).

#### 3.4.1 Oncology

Oncology represents a critical and growing burden across GCC countries. It is driven by increasing incidence rates, rising treatment complexity, and the high costs associated with advanced therapeutics. Cancer is now among the leading causes of morbidity and mortality in the region. This burden is compounded by demographic shifts such as ageing populations and lifestyle-related risk factors, including obesity and tobacco use (40). The treatment landscape has evolved with the emergence of novel interventions, particularly immunotherapies, targeted therapies, and gene-modified cell therapies. This enables more precise and effective options for cancer care. However, these innovations come at substantial costs, posing financial challenges to health systems striving to ensure equitable and timely access. The introduction of VBH and MEA, such as risk sharing and outcome-based reimbursement models, offers a pathway for balancing clinical innovation with financial sustainability (41–44). All GCC countries bear a substantial economic burden due to the provision of free cancer care, underscoring the need for a robust framework to assess, register, and approve novel oncology treatments. Accelerating patient access, integrating patient-reported outcomes, and implementing value-based cancer care are critical to ensuring sustainability and improved health outcomes (17).

However, there are significant barriers to its implementation. This includes fragmented health infrastructure, variability in outcome measurement standards, limited local real-world data, and challenges in integrating VBH frameworks within existing procurement and reimbursement mechanisms. Moreover, programs such as compassionate use, though vital for patients with rare or advanced cancers, face ethical, regulatory, and logistical complexities that hinder broader adoption (45–48).

#### 3.4.2 Immunology

The rising prevalence of autoimmune conditions such as rheumatoid arthritis, Crohn’s disease, and psoriasis presents a growing challenge for health systems in the GCC, particularly given the chronic nature and high treatment costs of immunologic therapies (49, 50). Targeted treatments like Janus Kinase (JAK) and interleukin inhibitors offer improved efficacy with reduced systemic toxicity but come at a significant economic burden. Biosimilars provide a viable, cost-effective alternative to originator biologics, delivering comparable clinical outcomes and improving key health economic indicators (51). The adoption of biosimilars is increasing, driven by multiple factors such as regulatory reforms that streamline approval processes and the lowering of development costs, which encourage more market entry. The authors note that despite initial challenges, the case for broader biosimilar use is strengthening, and a significant market shift is imminent as prices continue to drop and market expansion occurs (52).

To facilitate VBH implementation in immunology, it is essential to align reimbursement policies with clinical guidelines, engage stakeholders early, communicate benefits transparently, and support switching policies with regulatory incentives (53). These measures are crucial to ensuring that innovation translates into broad and equitable patient access, supporting both fiscal sustainability and improved health outcomes across the region (23, 54).

#### 3.4.3 Neurology

Neurological disorders are a leading cause of disability and the second leading cause of death globally, with rising prevalence in the GCC (55). Diseases like Alzheimer’s disease (AD) and multiple sclerosis (MS) strain healthcare systems, as current therapies offer limited benefits or high-cost disease modification (56–59). Although specialty drugs, including monoclonal antibodies and neuroprotective agents, show promise, adoption is hindered by low investment, regulatory barriers, and limited reimbursement (60–63). Advancing VBH in neurology will require targeted policies, RWE generation, affordability programs. It will also depend on payer engagement to align innovation with outcomes (61–64).

#### 3.4.4 Rare diseases

The burden of rare diseases in the GCC countries is significant. It is largely driven by sociocultural factors such as high rates of consanguinity and large family sizes. This contributes to the elevated prevalence of genetic disorders (65). A retrospective case–control study on 112 pregnant women with sickle cell disease (SCD) was conducted in Bahrain. The findings reveal significantly increased maternal and fetal risks associated with the condition (66). Public awareness of SCD in Bahrain was widespread, but critical gaps remained in understanding its genetic inheritance and prevalence (67). In Qatar, whole genome studies have identified founder mutations specific to certain ancestries, underlining the value of population-specific genomic data (68). Similarly, the UAE reports over 1,365 unique gene variants, many likely pathogenic, underscoring the genetic complexity within its population (69). Oman’s carrier rate for SCD and thalassemia combined is around 6% (70). Collectively, these data reflect a high regional burden of rare diseases requiring long-term, resource-intensive care.

Despite increasing recognition of rare diseases, multiple challenges hinder the effective implementation of VBH in rare diseases. Diagnostic delays, limited access to genetic testing, and fragmented care pathways compromise early identification and management (71). The lack of centralized registries and the minimal availability of RWD impede outcome tracking and value-based reimbursement planning. Furthermore, affordability remains a concern due to the high cost of orphan drugs, which often lack reimbursement clarity and depend on *ad hoc* financing mechanisms (68, 72–74). Although the SFDA has included incentives like priority review and fee waivers, the lack of long-term outcome evaluation tools remains a barrier to full VBH integration (75).

Gene therapy offers a novel and potentially long-lasting solution for genetic disorders by directly addressing the underlying genetic defects. As a one-time treatment, it aims to repair or replace faulty genes, with the potential to restore normal function and alter the course of disease (76, 77). FDA-approved cell and gene therapies vary in cost-effectiveness, with some offering value despite high initial costs (78). However, progress in rare disease applications is limited by regulatory, financial, and market barriers. This underscores the need for sustainable models that reflect disease burden, existing treatments, and economic feasibility (79, 80).

### 3.5 Foundational pillars of the GCC blueprint

**Figure 4:**
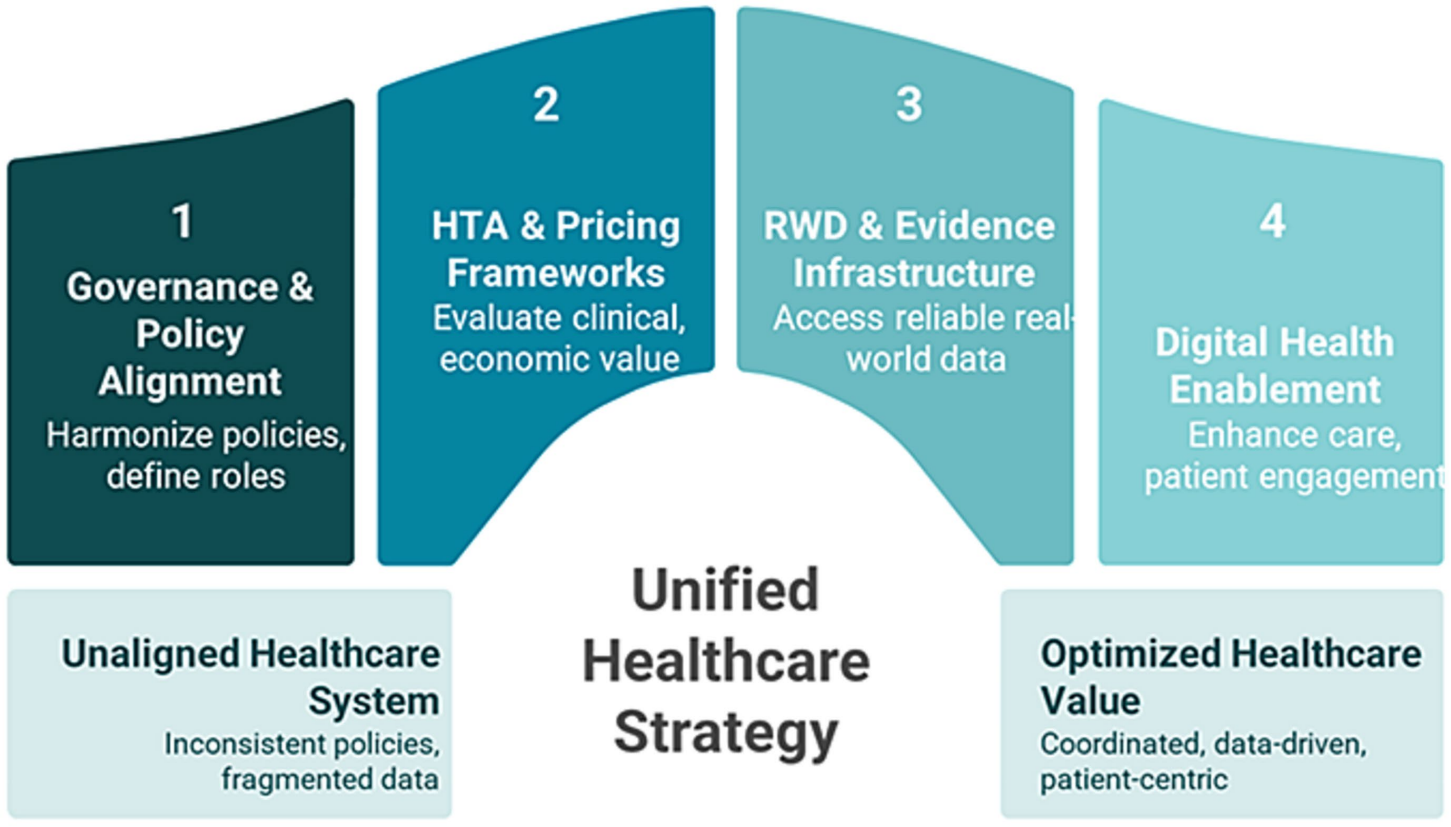
Four pillars of GCC value blueprint.

The GCC value blueprint is anchored in a commitment to equitable, sustainable healthcare through value-based care principles. Figure 4 depicts the four foundation pillars of the GCC value blueprint (31, 81–84).

#### 3.5.1 Governance and policy alignment

While countries like the KSA and UAE have made notable progress, bureaucratic inertia and budgetary constraints continue to hinder public payer adoption (85). Despite universal healthcare coverage, resource allocation disparities, especially between citizens and expatriates, persist (2). Tools like MCDA offer a pathway to ethical and transparent decision-making (86, 87). Integrating ethics review boards and patient advocacy will further strengthen policy credibility and inclusiveness (87).

#### 3.5.2 Health technology assessment and pricing frameworks

HTA infrastructure remains nascent in many GCC countries, with key limitations including insufficient local epidemiological and outcomes data, low HEOR publication rates, and limited professional capacity (40). These barriers delay VBH-aligned reimbursement decisions. Addressing these gaps will require investment in local research, cross-sector collaboration, and workforce development. Moreover, traditional pricing models often undervalue treatments in rare diseases and oncology due to unfavorable cost-effectiveness thresholds (88). Innovative approaches such as risk-sharing agreements (RSAs), value-based pricing (VBP), and patient assistance programs (PAPs) offer pragmatic alternatives (42, 89, 90). The SFDA has made strides by offering a structured orphan drug designation pathway, including incentives like regulatory support and fee waivers (26). Standardizing such frameworks regionally will be key to sustainable access.

#### 3.5.3 Real-world data and real-world evidence frameworks

RWD and RWE are critical enablers of VBH, especially in areas where clinical trial data are limited, such as rare diseases and oncology (25, 91–93). RWE supports regulatory compliance, post-marketing surveillance, and value-based pricing models. However, adoption in the GCC is challenged by infrastructural gaps, data privacy concerns, regulatory ambiguity, and limited technical expertise. Building effective RWD/RWE frameworks will require robust digital health systems, data governance policies, and stakeholder collaboration. Integrating local registries, especially for rare diseases, and capitalizing on the existing digital capabilities in the KSA and UAE will be essential to generating actionable evidence (25).

#### 3.5.4 Digital health enablement

Digital infrastructure is pivotal to scaling VBH in the GCC. Advanced systems in the KSA, UAE, and Qatar support outcome tracking, bundled payment models, and integration of patient-reported outcomes (25, 94). In oncology and rare diseases, digital platforms facilitate registry development, real-time monitoring, and streamlined access to clinical trials (95). They also enable remote care delivery and administrative automation, enhancing both system efficiency and equity of access.

#### 3.5.5 Managed entry agreements

**Figure 5:**
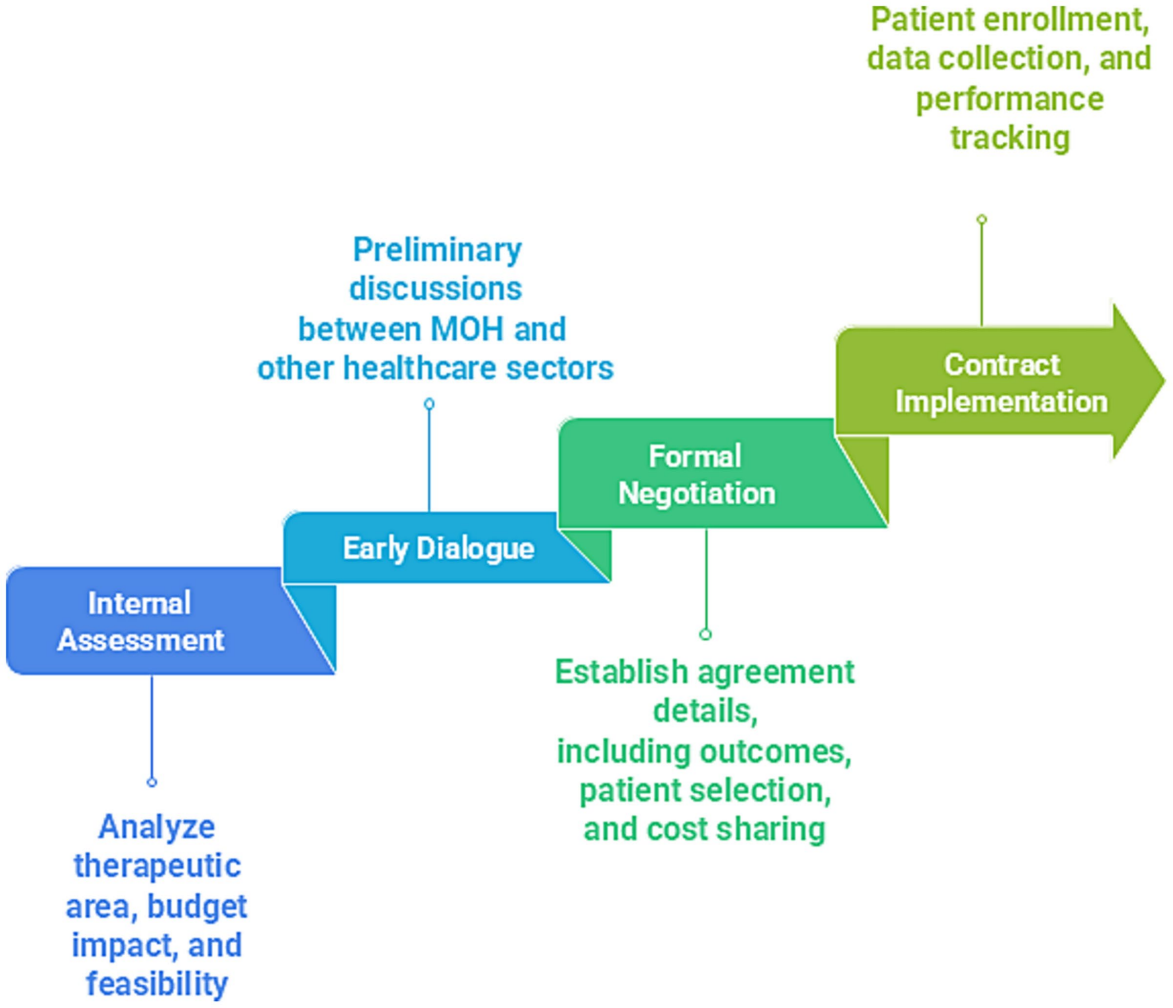
The four phases of MEA implementation.

MEAs are structured arrangements between pharmaceutical companies and payers or regulators that facilitate access to high-cost, innovative therapies while managing clinical and financial uncertainties. These agreements are increasingly relevant in GCC as countries adopt VBH models. MEAs can be classified as performance-based, linking reimbursements to specific clinical outcomes. They can also be financial-based, incorporating mechanisms such as price discounts or caps to mitigate budgetary impact (96, 97). Despite their promise, implementation faces several challenges in the GCC, including limited local outcome data systems, and a shortage of pharmacoeconomic expertise. Additional barriers include the absence of standardized frameworks, and cultural resistance to alternative reimbursement models. Figure 5 shows the four phases of MEA implementation.

MCDA is gaining traction in the GCC as a structured tool to support transparent, value-based healthcare decisions. It incorporates factors beyond cost-effectiveness, including clinical impact, equity, and patient preferences (98). In the KSA, researchers demonstrated the feasibility of implementing MCDA to establish a national evaluation framework, and stakeholders endorsed its role in enhancing transparency (99). Traditional HTA, while essential, may inadequately capture the nuances of rare diseases and oncology. In these areas, small populations and uncertain outcomes complicate standard evaluation models (89). In response, UAE researchers developed a tailored MCDA tool for orphan drugs, incorporating 10 weighted criteria to guide reimbursement decisions. These criteria include patient age, indication uniqueness, household burden, clinical evidence strength, disease rarity, budget impact, disease severity, therapeutic alternatives, health gain magnitude, and cost-effectiveness (100). Similarly, to address rising pharmaceutical costs and enhance transparency in purchasing decisions, Kuwait developed an MCDA tool. It was co-created by a diverse group of stakeholders, including pharmacists, regulators, and academics, through a structured seven-step process. The initiative aimed to support more consistent, value-based decisions, ultimately improving the sustainability of Kuwait’s healthcare system (101).

Key considerations for adapting MCDA frameworks in the region include addressing limited long-term clinical data, small patient populations, ethical dilemmas, and the need for stakeholder-inclusive decision-making (102) (Table 1).

#### 3.5.6 Access and affordability models

Early diagnosis plays a pivotal role in improving outcomes for rare diseases and cancers. GCC countries are investing in genomic programs such as the Saudi Human Genome Program, UAE Genomics Council initiatives, and the Qatar Genome Program (103–105). Additionally, expanded newborn screening and national cancer screening initiatives across GCC nations support timely detection (103, 105). However, limitations in HTA frameworks and data infrastructure hinder the integration of these tools into reimbursement systems (106).

#### 3.5.7 Risk-sharing agreements

RSAs provide a pragmatic solution to the uncertainties in real-world effectiveness and cost of high-priced therapies, particularly in oncology and rare diseases. They include performance-based models connected to clinical outcomes and financial-based models involving discounts or refunds (97). The KSA is actively adopting RSAs as part of its Vision 2030 health reform, offering a mechanism to balance patient access with fiscal sustainability (107). By adapting global models, the KSA can establish robust RSA frameworks tailored to local needs and regulatory conditions.

## 4 Discussion

The global healthcare systems are increasingly embracing value-based care models. The GCC region is actively adapting these principles into context-specific strategies to address its unique healthcare challenges and reform objectives. Vision 2030 marks a significant transformation in the KSA approach to healthcare, aiming to reshape the system through comprehensive reforms. This initiative seeks to modernize medical infrastructure, integrate advanced digital technologies, and enhance the skills of the healthcare workforce. It also aims to improve public health initiatives, and reform insurance models, as well as regulatory oversight. With a strong emphasis on elevating care quality and ensuring patient safety, these efforts aim to position the KSA healthcare system among the most efficient and accessible in the world (108).

Value-based care approaches aim to curb rising specialty drug costs by prioritizing patient outcomes and promoting responsible use (10). By linking reimbursement to clinical benefits, VBH fosters a more efficient healthcare system and aids in managing overall healthcare expenditures. The focus is on achieving optimal patient outcomes relative to the resources invested. This is particularly crucial in specialty care, where treatments are typically both expensive and prolonged. By connecting reimbursement to tangible health results rather than the volume of medication administered, VBH encourages the adoption of therapies that deliver clear and demonstrable benefits (109, 110). One of the most notable advancements in the shift from traditional fee-for-service models to VBH is the implementation of alternative payment models, such as Diagnosis-Related Groups (DRGs). These models incentivize providers to prioritize quality and efficiency over the sheer volume of services rendered. The goal is to reduce unnecessary procedures, optimize resource allocation, and enhance clinical outcomes (111).

In addition, digital transformation is also a cornerstone of VBH implementation across the GCC. Governments and healthcare institutions are investing heavily in electronic health records, telemedicine, and advanced data analytics platforms to support evidence-based decision-making and coordinated care (112–114). The digital infrastructure facilitates the continuous monitoring of clinical performance and patient progress, enabling timely interventions and improved care continuity. Population health management is a key priority, especially in addressing high-burden conditions such as diabetes and cardiovascular disease. By utilizing predictive analytics and stratified care approaches, healthcare systems can effectively target high-risk groups with preventive services and chronic disease management programs. This ultimately reduces long-term costs and enhances health outcomes. These investments not only support evidence-based decision-making but also foster greater transparency and case coordination (115, 116).

In the same vein, stakeholders’ engagement is equally important for driving cultural adaptation and achieving VBH’s success. Policymakers, healthcare professionals, and patients are actively involved in shaping VBH frameworks that are aligned with the region’s unique sociocultural and regulatory contexts (117).

GCC nations are also increasingly integrating patient-related outcome measures (PROMs) to ensure that healthcare delivery aligns with the values and preferences of patients. By incorporating PROMs into clinical pathways, healthcare providers can gain real-time insights into treatment effectiveness and patient satisfaction. This enables a more personalized and outcome-driven care experience (118). Additionally, the GCC is exploring innovative approaches such as stakeholder collaboration and servant leadership. The objective is to develop healthcare models that are not only effective but also culturally sensitive and socially sustainable (119). As the region continues to advance, these initiatives position the GCC on a promising trajectory toward building resilient, patient-centered health systems. These systems are designed to deliver high-quality care in an economically viable manner.

The significant growth of healthcare infrastructure in the KSA reflects a larger global trend seen worldwide (108). The KSA’s VBH implementation reflects a synthesis of the best global practices and innovative strategies tailored to its national context. Through digital integration, AI adoption, standardized care protocols, and collaborative partnerships, the country is forging a path toward a more efficient healthcare system. The model is also structured to be equitable and patient centered. To ensure consistency and quality in care, the KSA established the National Guidelines Centre, promoting the use of evidence-based practices across the healthcare system. This initiative aligns with global trends emphasizing standardized care protocols to reduce variability and enhance patient outcomes (120).

Countries like the United States, the United Kingdom, Sweden, and Singapore have each developed distinctive models to advance value-based healthcare, reflecting their unique healthcare landscapes and priorities. The KSA approach aligns closely with these global examples while addressing its healthcare challenges and opportunities. For instance, like the U.S. CMS Oncology Care Model, the KSA emphasizes performance-based incentives and coordinated care to improve outcomes (121). Its commitment to evidence-based clinical guidelines mirrors the UK’s NICE Framework for rare diseases. This strikes a balance between clinical effectiveness and social value (122). The KSA’s investment in digital health infrastructure promotes cross-regional collaboration, aligning with Sweden’s integrated care systems that unify municipal and county services (123). Meanwhile, although Singapore operates a multi-payer healthcare financing system, the KSA pursues diversification of funding through public-private partnerships. This approach aims to strengthen service delivery and financial sustainability (124). This indicates the KSA’s strategic capacity not only to adopt but, also to tailor established global healthcare models. This drives the country towards innovative and context-specific solutions that effectively address unique healthcare challenges. This approach accelerates the transformation toward a more efficient, patient-centered, and sustainable healthcare system.

In GCC, economic evaluations are limited by the absence of unit cost data, utility value sets, and defined cost-effectiveness thresholds. This prevents robust HTA and evidence-based decision-making (125). Coordination and policy implementation across GCC countries remain hindered by persistent fragmentation within healthcare systems. The absence of unified regional governance for health further compounds these challenges. Each country operates distinct delivery and financing channels often split across ministries (e.g., health, defense, and interior) and special agencies. This sometimes results in duplication of services, gaps in digital integration, and barriers to seamless patient management. Variable reimbursement schemes amplify these concerns, as payment structures and benefit packages differ not only by country, but by employer or agency. This limits regional mobility and data sharing. Constitutional guarantees of citizens’ rights to healthcare in most GCC states complicate efforts to introduce resource rationing or cost-effectiveness thresholds. These legal entitlements create barriers to restricting access or prioritizing therapies based on economic value. This results in frequent legal and public challenges, causing significant delays in the adoption of new technologies and evidence-based coverage policies. It also underscores the need for more integrated governance and harmonized health policies across the region (2, 85). Furthermore, insufficient integration of health economic evaluations into regulatory and reimbursement processes hinders progress. As a result, long-term recommendations struggle to move from pilot phases into sustained national policy. Suboptimal coordination between academia and health decision-makers reduces the translation of research into regulatory action and policy guidelines (125). Existing policies in GCC countries vis-a-vis challenges are presented in Table 2.

**Table 2:** Existing policies in GCC countries and their associated challenges.

Country	Existing Policy	Challenges
Kingdom of Saudi Arabia (133–136)	National Malaria Drug Policy Saudization Policies for Health Professionals National e-Health Strategy, non-communicable disease (NCD) Prevention Strategy Compulsory employment-based health insurance (CEBHI)	Limited integration of preventive and screening programs Health workforce shortages, fragmented private sector reforms
UAE (137, 138)	National Policy for Promotion of Mental Health National Policy on Vaccinations, National Drug Policy National Autism Policy National Policy to Promote Healthy Lifestyle	Challenges in full implementation of health data infrastructure Unified policy enforcement
Oman (139)	National Health Policy	Shortage of local health professionals Insufficient organizational alignment of NCD programs
Qatar (140)	National Health Strategy (2024–2030)	Limited digital health integration, weak innovation ecosystem Gaps in emergency preparedness Fragmented food safety oversight
Bahrain (141)	National Health Plan, “Sehati” National Health Insurance Program National Strategy for Control and Prevention of NCDs National Genome Program	Future focus on digital health One Health, and prevention Challenges remain with high NCD burden, weak surveillance, financial pressures, and unequal access.
Kuwait (16, 142)	National Strategy for the Prevention and Response to NCD and Mental Health Gulf Executive Plan to Combat Diabetes The Kuwait Cancer Control and Center Strategic Plan	Unstable leadership Weak data use, and poor policy continuity Preventive care is undervalued Private sector under-regulated, and systems for monitoring, limited financing.

VBH offers the promise of improved health outcomes by aligning healthcare delivery to measurable patient benefits rather than service volume. This framework advances the efficient use of healthcare resources and incentivizes evidence-based, high-value interventions. However, VBH is not without challenges. One of its key weaknesses lies in the difficulty of consistently measuring outcomes across varied clinical contexts. Additionally, implementing VBH models requires complex coordination among stakeholders and strong digital infrastructure. This can be resource-intensive and slow to establish.

Ethical dilemmas remain unresolved as GCC countries drive towards VBH, especially regarding citizen versus expatriate access and the prioritization of rare versus common diseases (2, 8). Legislative frameworks in many GCC states guarantee citizens universal health coverage. Yet, the extension of such rights to expatriate populations is more limited, leading to clear disparities and debates over entitlement and willingness to pay (2, 8). Policymakers face difficult decisions in allocating resources between common chronic conditions and high-cost, low-prevalence rare diseases. These decisions are often made without formal prioritization strategies that sufficiently address equity or social preferences (126, 127). Individuals with rare diseases may be disadvantaged despite the severity or lack of alternative treatments (21, 127). These unresolved ethical challenges complicate efforts to achieve truly equitable and sustainable healthcare outcomes under value-based systems in the region (2, 8).

In addition, the inclusion of patient advocacy groups and civil society organizations can help ensure that ethical principles such as distributive justice and equity are embedded in decision-making. MCDA, already piloted in KSA and UAE, provides a structured mechanism to incorporate ethical dimensions. These include disease severity, patient preference, and social equity in reimbursement decisions.

### 4.1 Policy recommendations

**Figure 6:**
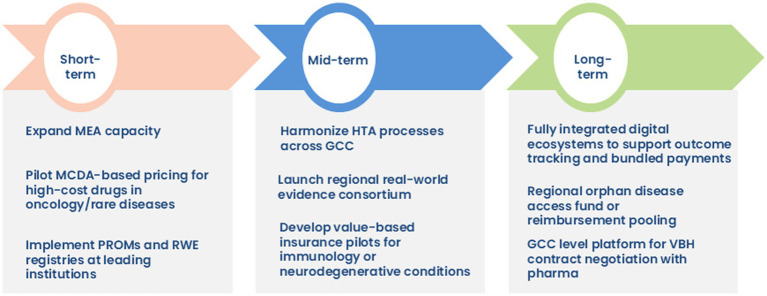
Roadmap to the implementation of VBH.

To accelerate the adoption of VBH across the GCC region, particularly in high-cost therapeutic domains such as oncology, rare disease, neurology, and immunology, a phased and strategic implementation roadmap is proposed. This roadmap emphasizes actionable goals across short-, mid-, and long-term timelines, prioritizing governance, RWE generation, digital infrastructure, and regional policy alignment (Figure 6).

#### 4.1.1 Short-term priorities

In the initial phase, efforts need to be directed toward expanding MEAs in the KSA and across the GCC to improve access to high-cost innovative therapies. Clearly delineated roles across regulatory bodies and procurement entities are essential; the consumer health informatics (CHI) can drive evidence generation, the SFDA may oversee regulatory compliance. The pilot implementation of MCDA frameworks, particularly for high-cost oncology and rare disease medications, will enable transparent and value-oriented pricing mechanisms. Concurrently, integrating PROMs and establishing institutional RWE registries can facilitate patient-centered care and digital health transformation.

#### 4.1.2 Mid-term priorities

Over the medium term, the focus shifts to harmonizing HTA processes across the GCC to enable consistent, evidence-based decision-making. Establishing a regional RWE consortium aims to address current gaps in data quality, governance, and regulatory harmonization, while fostering inter-country collaboration. Additionally, value-based insurance models can be piloted for chronic, high-burden therapeutic areas such as autoimmune and neurodegenerative conditions.

#### 4.1.3 Long-term priorities

Long-term strategies include developing digital ecosystems capable of supporting outcome-based payment models and real-time tracking of bundled healthcare services. A GCC-wide orphan disease access fund can be instituted as part of long-term initiatives to mitigate inequities in access to ultra-rare therapies. The creation of a unified regional platform to negotiate value-based pricing contracts with pharmaceutical manufacturers is recommended, supporting scale and economic sustainability.

#### 4.1.4 Strategic enablers for specialty therapies uptake

In parallel, several cross-cutting strategies may be implemented to enhance the uptake and equitable access of specialty therapies throughout the GCC region (60, 63, 64, 128, 129):

##### 4.1.4.1 Strategic partnerships and licensing

Public-private and cross-border collaborations between local biotechnology firms and global pharmaceutical manufacturers can facilitate co-development, technology transfer, and regional licensing. These mechanisms are crucial for scaling production and accelerating market entry of specialty therapies.

##### 4.1.4.2 Policy alignment

Reimbursement policies should be aligned with contemporary clinical guidelines to ensure broader patient eligibility and evidence-based coverage decisions. Policymaker engagement is critical for streamlining regulatory pathways. It is also important for supporting innovative pricing models, such as value-based pricing and outcome-linked contracting.

##### 4.1.4.3 Stakeholder engagement and communication

Building multi-stakeholder consensus, bringing together regulatory authorities, healthcare providers, payers, and patient advocacy groups, is imperative. Such collaboration is key to realizing the promise of VBH frameworks as an effective solution. Culturally sensitive and accessible communication tools such as lay summaries and multilingual patient materials can mitigate hesitancy, especially around biosimilars.

##### 4.1.4.4 Patient support and affordability programs

The implementation of co-payment assistance mechanisms, centralized patient hub services, and treatment navigation support systems can significantly reduce financial and logistical barriers. These measures also promote better therapy adherence and continuity of care.

##### 4.1.4.5 Digital health and RWE platforms

Leveraging digital platforms to enable real-time remote monitoring, treatment adherence tracking, and RWE generation is also considered critical. These tools support regulatory approval, payer decision-making, and personalized healthcare delivery.

##### 4.1.4.6 Access-driven policy implementation

The uptake of biosimilars can be enhanced by introducing regulatory incentives, integrating biosimilars into national formularies, and reinforcing their role within centralized procurement frameworks.

Although GCC-wide initiatives such as a unified pricing platform or orphan disease access fund are strategically desirable, their feasibility will depend on phased implementation and pilot projects. Lessons from regional precedents, such as the Gulf Joint Procurement Program and the Abu Dhabi HTA roadmap, demonstrate the need for iterative scaling, strong governance, and cross-border coordination (27, 30). Positioning these strategies as pilots with defined milestones may increase political and operational viability across diverse GCC systems.

## 5 Limitations

This study employed a consensus-based approach, which provides valuable exploratory guidance derived from expert perspectives. This methodology is well suited for synthesizing diverse stakeholder insights in areas where empirical evidence may be limited. However, the findings should not be interpreted as definitive or generalizable evidence. Rather, the recommendations should be viewed as preliminary consensus-based guidance intended to inform future policy, practice, and research directions. As with all consensus-driven work, the potential for subjectivity and contextual bias exists. This underscores the need for future validation through empirical studies, real-world implementation, and longitudinal evaluation to establish the robustness and applicability of these conclusions.

## 6 Conclusion

As healthcare demands continue to evolve, the GCC region finds itself at a pivotal juncture where traditional volume-based care approaches are inadequate for addressing increasing complexities and costs of advanced medical conditions. The growing reliance on sophisticated treatments necessitates a shift towards a sustainable and results-oriented model for financing and delivering care. VBH emerges as a promising exploratory pathway to better align healthcare spending with patient outcomes. It also fosters more equitable and efficient use of costly therapies.

In this context, the KSA is taking significant strides to enhance specialty care, particularly in oncology, neurology, immunology and rare diseases. The country is committed to improving access to cutting-edge treatments, and expanding specialized healthcare facilities. It is also investing in workforce capabilities to meet rising demands for complex and intensive medical services. These initiatives not only aim to enhance patient outcomes but also to build a resilient healthcare system capable of adapting to future challenges.

However, the implementation of VBH in GCC still encounters challenges, including issues related to data interoperability, resistance to change among providers, and the need for workforce upskilling. Nonetheless, the momentum towards reform is evident. Through regional policy alignment, digital investment, and stakeholder collaboration, the GCC is laying the groundwork for a patient-centered, efficient, and value-driven healthcare system.
